# Framework for Integrating Productive, Contributory, and Noncontributory Work with Safe and Unsafe Acts and Conditions

**DOI:** 10.3390/ijerph20043412

**Published:** 2023-02-15

**Authors:** Xavier Brioso, Claudia Calderon-Hernandez

**Affiliations:** GETEC Research Group, Department of Engineering, Pontifical Catholic University of Peru, Av. Universitaria 1801, Lima 15088, Peru

**Keywords:** productivity measurements, health and safety measurements, last planner system, health and safety management, workers

## Abstract

It is common for companies that are in the process of implementing the Last Planner System (LPS) journey to attempt an increase in productive work and a reduction in waste, such as contributory and noncontributory work. Even though the LPS has proven to have a synergy with the health and safety requirements, companies with deficient health and safety management systems tend to classify work involving substandard acts or conditions as standard, and then pretend to benchmark against other companies that are indeed performing safe work. The following work introduces a framework to simultaneously register and analyze productive, contributory, and noncontributory work, with the substandard acts and conditions in a construction site, allowing for the measurement of production and health & safety indicators simultaneously. In the absence of technology that automatically captures these indicators, it is proposed that simultaneous measurements be made through direct inspections and photo and video recording by means of a handheld camera. The proposed continuous improvement framework follows the steps indicated below: (1) defining the productive, contributory, and noncontributory work with surveys performed on the most representative stakeholders of the industry; (2) proposing a new classification of production and safety work; (3) assessing the level of application of the LPS in the company; (4) measuring the indicators; (5) improving the use of the LPS and performing new measurements; (6) statistically linking deadly, serious, and minor accidents, standard and substandard acts, standard and substandard conditions, and productive, contributory, and noncontributory work. This framework was applied to a case study of a building project in Lima and the results were improved simultaneous indicators, especially the health and safety indicators. Automated classification of productive and nonproductive work using technology still represents a challenge.

## 1. Introduction

Lean construction can be defined as the adaptation of lean manufacturing or Toyota Production System (TPS) to construction projects, it is based on the participants’ collaborative planning while applying concepts and principles aimed at the reduction of losses (waste), the generation of value for the client and stakeholders, and the continuous improvement of processes and flows [1]. Lean manufacturing could not have been conceived without the prior development of a safe system, rooted in the 5S philosophy, and a drive for normalization and standardization, which obviously includes safety practices. From the beginning, the makers and followers of the lean construction philosophy, starting with Lauri Koskela, included these precepts, the 5S, standardization, safe work, etc. [2]; a mindset ingrained in developed countries and the best worldwide companies, regardless of their origin. It is inconceivable that companies intend to generate value, eliminate waste, and collaboratively plan different activities, without first ensuring a reliable system based on respect for human life.

The last planner system (LPS) is a flexible production planning system that integrates support areas and is designed to generate a predictable workflow and quick learning in all phases of a construction project [3]. LPS allows for the implementation of the lean construction philosophy [1].

There is evidence that proves that the last planner system integrates production, safety, and health in an optimum way, improving the indicators of direction and management, and the indicators of occupational accidents [4,5,6]. We can state that LPS has synergy with safety and health systems, which are compatible with OHSAS 18001:2007 standard “Occupational Health and Safety Assessment Series” [7]. In Peru, health and safety laws have a structure similar to that of the OHSAS 18001 [8]. Since 2018, the International Standards Office has replaced OHSAS 18001 with ISO 45001:2018 standard “Occupational health and safety management systems–Requirements with guidance for use” [9]. It is expected that LPS will also be compatible with ISO 45001 and Peruvian health and safety laws. These systems need an effective method to move from planning into implementation and operation, verification, and revision by direction. This method can come from the evident synergy that exists with LPS. The simultaneous measurement of productivity and safety indicators at minimum cost is advisable.

In emerging and third-world countries, it is common to find deficient health and safety management systems in construction companies. For example, in Peru, many companies begin the Lean Journey without first implementing a correct safety culture [10,11]. On the other hand, they do not even have official statistics such as safety indicators. The Peruvian Ministry of Labor only records the accidents, incidents, and illnesses reported by companies as some sort of affidavit, however, they are not connected to the number of workers or man hours [12].

Benchmarking means researching to find and apply the best practices of companies worldwide [2]. With questions such as: “how did this competitor achieve better indicators than our company? Is trying to access their good practices legitimate, without being considered industrial espionage?”. Companies with high standards have the good practice of measuring indicators to compare performance between different projects to compare themselves with their competitors, promoting continuous improvement [11,13]. Evidently, the strengths and weaknesses of the company must be previously evaluated.

Furthermore, the capture, integration, processing, and analysis of work data to measure productivity, performance, and work categories, among others, is a challenge for construction companies [14,15].

Work sampling (WS) is a technique used by researchers to define and understand the types of productive work of workers on project sites [16]. However, there are still variations and discrepancies between authors [17]. For instance, [18] defines indirect work as talking, preparation, and transportation, while [19] classifies them as preparation, work supplements, administrative, and unusual elements. WS helps the contractor to evaluate the productivity rate, identify the reasons for noncompliance, take corrective actions, reduce waste, and improve performance [20]. However, the measurement of WS indicators has not yet been considered simultaneously with safety indicators. The main purpose of this study is to present a framework that allows the measurement of productive, contributory, and noncontributory work with substandard acts and conditions simultaneously. These new definitions will allow benchmarking between companies. Also, the current state of technologies that allow the simultaneous measurement of these indicators is reviewed.

## 2. Production Systems

### 2.1. Work Sampling (WS) 

WS consists of performing on-site observations and analyzing their results to establish what the individual workers are doing during specific time frames [21,22].

Before 1985, WS studies adopted the classification of two categories: direct work (DW) and nondirect work. DW is related to value-adding work time [23]. However, the lack of consensus created various subcategories of nondirect work [17]. After 1985, most researchers applied two additional categories, supportive work and waste time, which eventually evolved into indirect work (IW) and waste work (WW) [17,23]. IW could be defined as necessary and supportive work for DW. WW is a work that is not necessary [23]. In Latin America, the use of the productive work (Direct Work), contributory work (indirect work), and noncontributory work (Waste Work) categories is deeply rooted [24]. In this study, Latin American denominations will be used because they are commonly applied in our work (productive work (PW), contributory work (CW), and noncontributory work (NCW)).

The activities are registered onsite through videos and photos for posterior analysis using WS [22]. This approach allows measuring the level of activity in an operation [24], providing a snapshot of the circumstances in which the measurements were performed [22]. Using a representative sample large enough to be statistically sound, it is possible to predict a specific characteristic in an element within a project, or the project as a whole. Even though the prediction is not exact, the results are accurate enough to simulate the real situation, analyze it, and take corrective actions. It is important for the sample to have the following consistency characteristics: (1) the condition of each inspected unit must be independent of the conditions of the other units; (2) each unit must have the same probability of being selected; (3) the basic characteristics of the batch selected for sampling must remain constant [22].

The method of proportion estimation is used to measure the degree of certainty of the sampling process since the obtained results can be expressed as proportions. According to [24], the probability of occurrence of an event can be estimated using Bernoulli’s sequence, as a proportion of the occurrences of said event, in which *X*1, *X*2, …, *XN*, are *N* independent tests, and each *Xi* is a random variable that can take the value of one when the event takes place, or zero when the event does not take place, in test *i*. Thus, the parameter *P*, corresponding to the probability of occurrence of the event in a test, can be calculated using Equation (1).

(1) $$P=\frac{1}{N}\sum_{i}Xi$$

According to the central limit theorem, in which for a large *N*, *P* has a normal distribution, and from the confidence interval, the range of error on each side can be calculated using Equation (2) [24]. Statistically, the sample can be validated from three concepts: confidence level, margin of error, and proportion per category. The first one provides the reliability of the result, the second one gives the accuracy of the estimated value, and the last one supplies the expected proportion in the sample. In other words, how the sample responses are distributed. The number of samples for the required conditions can be calculated using Equation (2) [22].

(2) $$N=k^{2}\frac{P\left(1-P\right)}{L^{2}}$$

where:

*L* = range of error on both sides; 

*N* = number of tests (observations);

*k* = value of the standard normal variable for a confidence level.

The expected distribution between productive and nonproductive work (direct and nondirect work) is 50:50. Similarly, it is considered acceptable to have a level of confidence of 95%, and a margin of error of 5% to represent the work distribution for an entire project. This can be achieved using 384 samples [22].

In different projects studied in Peru, the professionals in charge defined PW, CW, and NCW differently. Thus, the obtained measurement could not be compared [11]. In other words, the tasks must be defined in the same way to achieve benchmarking.

### 2.2. Crew Balance Chart 

Crew balance charts are the “man–machine charts” from industrial engineering, adapted to the construction sector [22]. They provide an effective way to show the relationship between the activities of the members of a crew, and the equipment they use. To make a balance chart, it is necessary to observe and measure the time used by each worker and machine, on each task of an activity. Ideally, times must be measured in several work cycles, to validate their accuracy and variation during the cycles [22,24]. The project activities are registered through videos and pictures using the Crew Balance chart [22].

### 2.3. Classification of the Production Work and Benchmarking 

Work performed by workers and equipment can be classified into three categories [24,25]: (1) productive work (PW): it contributes directly to production and generates progress; (2) contributory work (CW): it must be carried out so the PW may be executed; it does not generate progress, however, it is necessary. It also does not provide value for the client directly; (3) noncontributory work (NCW): it does not generate progress and it is not necessary; it has a cost and falls directly in the waste category.

It is essential to define each task as PW, CW, and NCW, and to ensure that these definitions are equivalent when benchmarking. For example, in Peru, there are different definitions for the same task, which yields erroneous conclusions when comparing companies [11]. There is no standard that defines each type of work. Thus, it is not possible for companies to benchmark against each other, since the classification of one activity can vary from company to company, or even between projects. What is considered CW, could be considered NCW in another company, and so on. The need to define a standard is established, so that benchmarking is possible as they do in first-world companies.

It is common for companies beginning the lean journey to attempt an increase in productive work and a reduction in waste [26]. This approach boosts the productivity indicator, especially in activities with high incidence in cost, repetitive, critical, or with low productivity levels. The most commonly used sampling techniques to measure the PW, CW, and NCW percentages are by work sampling and a crew balance chart [22,24]. 

### 2.4. Proposed Survey to Benchmark the Types of Activities 

The sample size was determined based on [27]. Equation (3) determines the size of the sample n based on the following parameters: *Z* = 1.96, corresponding to the number of standard deviation of the normal distribution based on the level of significance adopted of 95%; the universe size *N* was the number of building projects built [28]; *ε* = 5%, is referred to the maximum error acceptable; and *p* = 50%, considering that there were no previous estimations for none of the selected definitions [27,28].

(3) $$n=\frac{Z^{2}\cdot p\cdot \left(1-P\right)\cdot N}{\left(N-1\right)\cdot \varepsilon^{2}+\left[Z^{2}\cdot p\cdot \left(1-p\right)\right]}$$

### 2.5. Last Planner System (LPS) 

LPS was developed by Glenn Ballard [29], who stated: (1) planning should be considered as a system, and not based only on the skills of the professionals in charge of programming; (2) the performance of the planning system must be measured; (3) errors in programming must be analyzed, the root causes of these errors must be identified, and corrective measures must be adopted, then results must be evaluated [30]. LPS states that the further the prediction, the more inaccurate it will be [29], so the system gives the following recommendations: (1) during planning, the level of detail of the task should be increased as the date of its execution approaches; (2) planning in a collaborative manner with all project stakeholders, including support areas, such as health and safety, logistics, and quality, among others; (3) opportunely identifying constraints and enforcing their requirements to execute planned assignments as a team; (4) making reliable promises; (5) learning from the interruptions [3,29]. By this, the variability is reduced, and the activities are achieved more efficiently. The LPS elements are (1) master planning (master scheduling): deadlines and milestones are established in the general schedule, and a list of tasks is determined by selecting the construction processes according to the budget and supplies, labor, and available equipment [29]; (2) pull planning phase session: it is a meeting where all the areas involved in the execution of the project have to identify the “handoffs” to be done between all participants, meaning, they are part of the design of the different alternatives to the schedule. The sectorization consists of the team dividing the measurements of all the activities (processes) of one building in a number of sectors in order to create a balanced production line, with resources (workforce, equipment and machinery, and materials, among others) that can be executed in a workday and that enables the correct conditions of everyone involved [31]. All planners must identify the logistics among tasks by adjusting their sequential schedule. These agreements are as compromising as a contract [32]. The attendance and participation in these sessions must be agreed upon in the contracts with the subcontractors [33]; (3) look-ahead planning: the look-ahead plan is usually between two and eight weeks long for building projects and it must be developed and communicated so that everyone involved is aware of the activities scheduled [29]; (4) constraint analysis: when scheduling the activities in the look ahead, an analysis is done so that there are no impediments to its completion. This means it is free of constraints that might generate a breach in the flow, waste, and delays. The constraints can be defined as prerequisites for an activity that, if not covered on time, might produce delays in the production flow [11]; (5) weekly work planning (weekly programming): we must prioritize compliance with the first week of the lookahead, use buffers according to variability and complexity, and provide alternate tasks to execute in case of unforeseen events [29]; (6) daily programming: a very important reason to have a daily program is to make performance measurements, not just of the working crew, but of each of the personnel members, making sure if a worker is productive or not and evaluating if the person has the adequate tools, as well as checking which factors are influencing their productivity, such as health, weather, lack of water, bad eating habits, demotivation, lack of safety planning, etc. [10]; (7) learning (reliability analysis): measurement of the planning system’s performance with the percentage of plan completed (PPC): LPS measures the performance of the weekly plan through the completed task (assignment) percentage (PPC), which is the number of accomplished items divided by the number of programmed tasks (assignments) for any given week. The reliability analysis is the exercise through which we can measure the quality of the programming. Root causes that have hindered achieving the 100% fulfillment of the weekly plan (PPC) can be identified and attempts can be made to eliminate them [29]. 

In recent years, LPS has been implemented by some contractors in Peru, however, its full potential has not been developed yet [34].

## 3. Health and Safety Systems 

The leadership and participation of workers have become essential for health and safety management systems. For instance, the International Labor Organization (ILO) and the World Health Organization (WHO) urge their member countries to include workers as key participants in management systems in their regulations [35,36]. Coincidentally, in 2018 the ISO published ISO 45001 requiring companies to give workers a leading role in the review and approval of health and safety management systems, as a strategy to reduce and eliminate occupational accidents and illnesses. It is very important to include workers and other stakeholders in the planning meetings [9]. In the same sense, the *Agile Practice Guide* [37] indicates that lean thinking is a superset, sharing attributes with agile and kanban, modern methods that emerged in the mid-2000s that also promote teamwork to organize safe work areas. LPS is also a modern method focused on teamwork. Its structure is based on lean thinking [1] and is synergistic with safety management since it is based on respect for people [5]. Therefore, the inclusion of all stakeholders in these collaborative meetings cannot be postponed.

Additionally, a study determined that the project and firm-related factors are the most influential in promoting the effectiveness of health and safety training sessions among the success factors that promote health and safety performance. This group consists of variables, such as project type, project size, project duration, and firm size [38]. Consequently, it would be an excellent practice for companies dedicated to the execution of similar projects to benchmark by exchanging their good practices in health and safety training sessions.

Health and safety management systems are based on the evolution of the accident causation theory of Herbert W. Heinrich [7] and immediate causes, basic causes, and operational control failures are defined as the root cause of accidents [39]. In turn, the immediate causes can be classified as substandard acts and conditions. The basic causes can be classified as work factors and personal factors. In several countries, safety regulations are based on these concepts [7]. For example, in Peru, [40] it defines, amongst other concepts, the following: (1) personal factors: related to limitations in experience, phobias, and stress affecting the worker; (2) work factors: related to the work itself, as well as the work conditions and environment; (3) standard act: any safe action or practice executed by the worker; (4) substandard act: any incorrect action or practice executed by the worker; (5) standard condition: any safe condition in the work environment; (6) substandard condition: any condition in the work environment that may cause an accident.

In summary, two types of causes can be defined: due to the employer’s responsibility, and due to the worker’s responsibility. If, and only if, the employer has verified the personal factors of the work applicants, provided training and education to the workers, and has given them the proper personal and collective protection gear, accident causes could be considered exclusively as the worker’s responsibility. In any case, the workflow could be halted due to supervision orders, incidents, accidents, or illnesses. Workers’ behavior can be studied using different management tools and techniques [10,41,42]. For example, behavior-based safety (BBS), as its name indicates, considers the safe behavior of workers as the basis of health and safety management [43]. BBS aims at identifying and modifying the worker’s unsafe action by means of a combination of observation, feedback, training, and goal setting. In addition, BBS has an inverted pyramid approach where the role of the worker is fundamental [44]. We can state that BBS consists in measuring and analyzing the indicator of substandard acts and conditions. These are performed through site inspections with trained staff, able to determine how each worker is operating, and under which work conditions.

Also, there are company policies contractually accepted by their workers. Companies can include penalties for workers committing substandard acts in their internal regulations [45]. Obviously, these substandard acts generate waste in companies and production flow standstills. Not only the worker, the entire crew is involved, as well as the subsequent activities. If the company has not secured in their staff trained workers that can replace the offender, these acts can also generate work stoppages with severe financial waste.

As previously stated, the LPS and the health and safety systems are synergic since there exists evidence of improvement in the safety indicators when both systems are applied simultaneously. For this reason, our research is focused on projects that implement LPS.

## 4. Measurement of Productive, Contributory, and Noncontributory Work with Substandard Acts and Conditions Simultaneously

**Figure 1:**
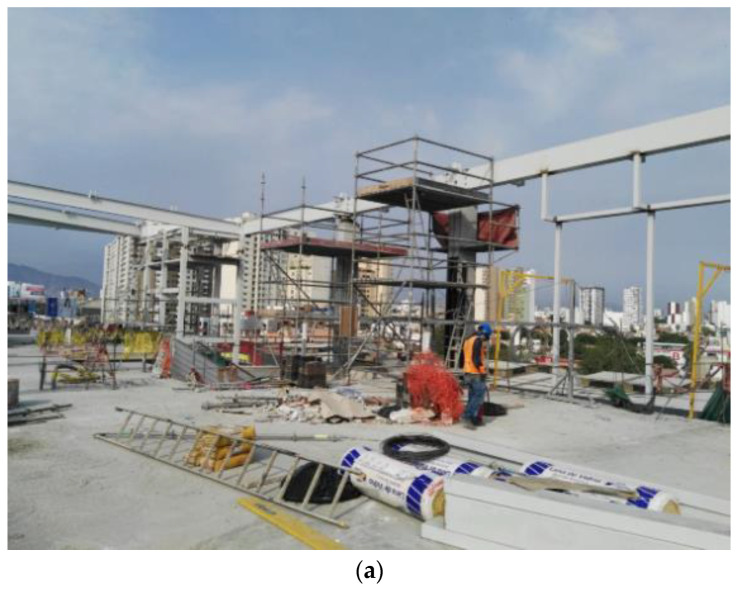

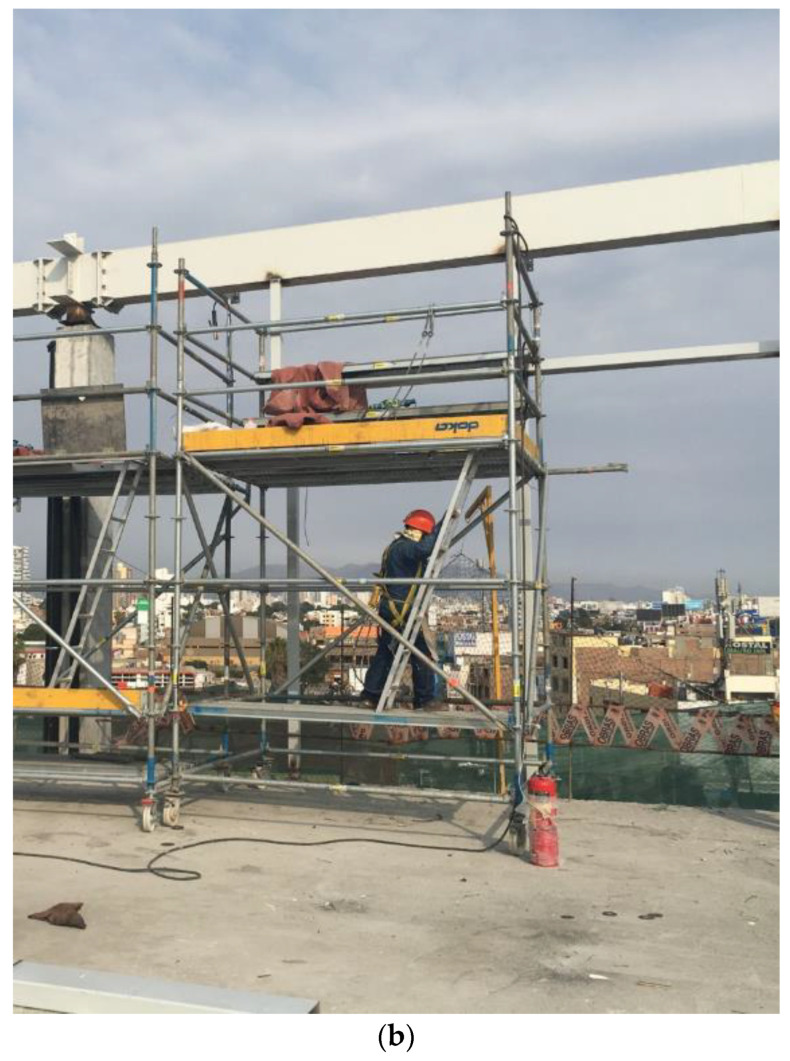
(**a**) Standing worker (NCW-SA-SSC); (**b**) Using scaffolding (CW-SA-SSC).

Due to the simultaneous record of work types, workers’ act types, and site conditions, there is a classification of production and safety work, as shown in Table 1 [11]. Evidently, productive, contributory, and noncontributory works are valid and comparable only when they comply with standard safety acts and conditions. On the other hand, there are nine work classes (numbered 2, 3, 4, 6, 7, 8, 10, 11, and 12) that can produce the aforementioned waste. These types of waste can pass undetected in companies with deficient safety systems, or even worse, be deliberately ignored in order to create false production indicators aiming to artificially increment their productive work. Figure 1 shows two examples of classified work.

Figure 1 shows two examples of work classification: (a) standing worker (NCW-SA-SSC): this action can be defined as noncontributory work since it is located on solid ground, it is a standard action, and as the surrounding work area is disordered, it can be defined as substandard conditions; (b) using scaffolding (CW-SA-SSC): this action can be defined as contributory work since it is located on a solid platform, it is a standard action, and as the surrounding work area is disordered, it can be defined as substandard conditions.

Additionally, in case of an accident or illness, this would have a financial impact that could, in turn, be subdivided into direct and indirect costs [46]. These direct and indirect costs can be defined as follows: (1) direct cost: expenses generated by the accident such as compensation payment, medical, pharmaceutical, and transfer expenses. This cost is easy to calculate since it is a percentage of the contribution received by each worker. It is paid as a company and employee contributions to the Work Accident Liability Insurance Associations, and they finance the compensations and other expenses; (2) indirect cost: expenses generated by the accident that are difficult to calculate, such as wage costs, extra expense due to increased staff management, material costs, expenses endured by the worker, expenses endured by the company, and expenses endured by society. Despite not having precise costs, it is possible to estimate comparative states of accident rates if the same system is used in all cases.

## 5. Simultaneous Recording

As previously stated, to apply the work sampling or crew balance chart tools, the tasks are registered through videos and photos for posterior analysis. Also, the safety inspections could be registered using the same technology. Additionally, when films or videos are analyzed, there is the advantage that the results of the evaluation can be reviewed, understood, and audited transparently by any stakeholder [22].

However, is it possible to automate the information processing according to the new classification of production and safety work? 

Computer vision and sensor-based technologies are mostly used by researchers, being able to automate data collection for work sampling and activity analysis, measure inputs, outputs, and cycle times, and monitor factors that can have an impact on the productivity and safety of workers [47].

The level of complexity of image processing increases as more people are involved in the construction process. Turaga et al. define two levels of complexity [48]: (1) actions: which are conducted by a sole person and are characterized by simple movement patterns, and (2) activities: which are actions coordinated and executed by small groups of people, and, therefore, they are more complex than an action [48]. This has not changed to this day; it is a technological application that is under development. According to Rao et al., vision-based technologies have had good results in health and safety management systems, detecting people who are close to hazardous areas, and supervising the conduction of safe work, among others [49]. In that sense, in a study developed by Khosrowpour et al., a vision-based technology system is used to detect the position of workers and classify their work with an average accuracy of 70% of the detected positions [50]. However, it is assumed that the position of the worker implies that they are doing a type of work, without distinguishing whether they are doing productive or nonproductive work, for example, standing around doing nothing. Detection of fine motion remains a challenge for video-based technologies. Pose estimation techniques are widely used in ergonomics studies, however, these still need to be improved to determine the categories of productive work [47]. Furthermore, there is a study in progress that analyzes the opportunities of combining data from geographically located observations of workers with data obtained from WS [51,52]. However, there is no further information on whether it could be implemented in real time. Automated classification of productive and nonproductive work using technology still represents a challenge [47,53].

The efforts described in the lines above are important, however, it has been determined that there is still a lot of work to be done to accomplish the automation of the measurements of productive, contributory, and noncontributory work, and further, the automation of these measurements including standard acts and conditions simultaneously.

According to this, this research study is mainly focused on the use of hand-held cameras as a method for capturing photographic and video material. The use of them is selected since there is no technology that can automatically identify and classify these types of work. This will allow us to subsequently review the information collected on site, to have exact and statistically valid measurements. In compliance with the law, the workers were asked by company executives and they agreed to be photographed and filmed. The company already used an inclusive collaborative method in its work, which supported this acceptance.

Since the intention is to use a simple and representative methodology to simultaneously measure production and safety, our proposal involves using work sampling and safety inspections. This will allow the registration and analysis of productive, contributory, and noncontributory work, as well as substandard acts and conditions, at the same time. Balance charts would imply larger efforts and more opposition towards implementation from the interested parties.

With this purpose, it is essential that the inspection staff is properly trained and educated on safety and production work classification. If the company already has a team trained to measure safety indicators, it would be convenient to prepare them for production, and vice versa. Also, the frequency of these simultaneous measurements would need to be decided. While the health and safety indicators are measured daily, the time dedicated to classifying productive work could generate delays, and therefore, additional general expenses. Therefore, the idea is to measure as little as possible and to maintain efficiency levels.

## 6. Proposed Methodology for Statistical Correlation between Accidents and Type of Work

Ever since Heinrich published his famous 300-29-1 model (300 Near misses and 29 Minor Injuries per 1 Major Injury) [54], many methodologies have been proposed to connect accidents and incidents [46]. Accidents occur due to human factors and mechanical and environmental factors, and more systemic research models are required [55]. However, the scope of this study considers the statistical information of the research already conducted and reported according to the methodologies promoted in the country of the case study. Making an analogy with the Heinrich model, we propose to link fatal, serious, and minor accidents, and the estimated man-hours of each type of work within a timeframe, for example, one year. Statistical correlations between occupational accident rates and the productive, contributory, and noncontributory work of the company can be simultaneously obtained work by work, or by accumulated work, investing the least number of resources and, therefore, using a more economical method. In addition, the quality of the information will be improved, since by making measurements with integrated indicators, the uncertainty of making measurements separately and with no standard methods, with greater deviations and, therefore, with higher costs, will be reduced. According to this, the proposed methodology for statistical correlation consists of:

Step one: representative work sampling in a project during a set timeframe, for example, one calendar year. Microsoft Excel is used to process this data.

Step two: collection of the cumulative percentages of each work type in the sampling.

Step three: estimation of the number of man hours assigned to each type of work within a certain timeframe, for example, one calendar year. According to regulations [40,56], all employers must record and report to the Ministry of Labor fatal, serious, and minor accidents, the number of workers, and the number of man hours per month, per year, etc. This study proposes that the cumulative percentages of each type of work be linked to the total man hours in the same timeframe, in order to calculate the man hours on each type of work. Microsoft Excel is used to process this data.

Step four: To link the fatal, serious, and minor accidents, and the estimated man hours on each type of work within a timeframe.

Step five: Calculate in a simple manner all the relations or indicators required, in addition to the conventional accident rates.

Step six: To build models similar to Heinrich’s to show the proportion of the different types of accidents and types of work.

## 7. Simultaneous Measuring Framework Proposal for Productivity and Safety

Construction project management systems can be compatible with each other by flexibly adapting sequences and processes, and combining their tools and techniques [57]. According to this, the following framework is proposed:

Step one: survey performed to benchmark the types of activities: Definition of the work performed by the workers according to the categories of productive, contributing, and non-contributing work. This definition is obtained through a survey performed on several experts on the subject. The survey design considers the described by [27,28].

Step two: choosing a project for the case study.

Step three: evaluating the level of implementation of the LPS on the study case project.

Step four: work sampling and simultaneous evaluation of work type and safety inspections and the design of the work sampling. Simultaneous evaluation of work type and safety inspections, assessing the work environment conditions and the type of acts of the workers. Video-recording of these acts to provide evidence of the unbiased evaluations required by this method. Microsoft Excel is used to process this data.

Step five: implementation of safety and production corrective measures: worker re-training after a substandard act. Change a substandard condition to a safe one. Analysis of the obtained results according to the new classification of production and safety work proposed in this paper. Introduction of the production corrective measures derived from this analysis and applying last planner techniques during the meetings to improve the indicators. Measuring the indicators based on the corrective measures.

Step six: statistical correlation of fatal, serious, and minor accidents and types of work: apply the proposed methodology. Microsoft Excel is used to process this data.

**Figure 2:**
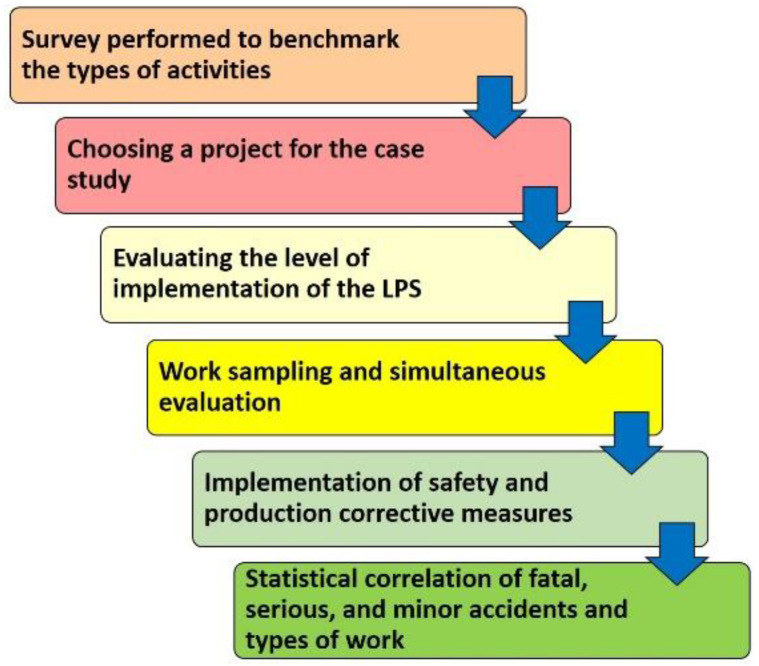
Research methodology.

Figure 2 shows the flowchart of the research methodology.

## 8. Results and Discussion

### 8.1. Survey Performed to Benchmark the Types of Activities 

The research universe was composed of civil engineers and architects that work in the construction of buildings of over five stories. Equation (3) determines the size of the sample, n, based on the following parameters: Z = 1.96 (number of the standard deviation of the normal distribution based on the level of significance adopted of 95%); the universe size N was the number of building projects built in Lima and Callao between August 2015 and July 2017 that have an elevator [58]; ε = 5% refers to the maximum error acceptable; and *p* = 50%, considering that there were no previous estimations [27,28]. After applying these parameters to Equation (3), the number of obtained interviews needed was 315 in the universe of 1738 projects. After verifying the integrity of the data, 334 surveys were performed, those interviewed were civil engineers or architects that worked on different study projects between August 2015 and July 2017 (Table 2). Each professional assessed had to classify a list of 128 activities in terms of productive, contributory, and noncontributory work. The result of this assessment was used as a guideline to standardize the classification of the activities. Although the profile of the respondents is optimal, similar scores could be obtained in some work classifications. As one of the objectives of this study is to do benchmarking, the researchers and the collaborating company agreed that the criteria to define the classification would be by simple majority.

The results of this survey were grouped into 46 types of activities as shown in the table below (Table 3). For example, the placement of vertical and horizontal reinforcement was grouped under the activity placement of materials.

The 128 activities can be used to analyze similar projects but for these case studies the summarized list of activities was chosen.

### 8.2. Choosing a Project for the Case Study

The case study belongs to a large real estate company with 18 years of experience building massive housing and office projects. Since 2011, it has been associated with the Lean Construction Institute based in Peru, and, therefore, it benchmarks with similar real estate companies, sharing its tools, techniques, and good practices, such as safety training strategies, which are essential for good performance in occupational accidents, according to [38]. In compliance with Peruvian law, the worker agrees that at any time during the investigators’ visit, the employee’s work may be photographed or videotaped by the researchers for research purposes.

This project was a 15-story residential building of 190 apartments, made of reinforced concrete. It was monitored through a hand-held camera which allowed for effective work sampling. The equipment used in the study was a Canon Powershot A2300, with a 16.0 MP Image Sensor, DIGIC 4 Image Processor, 5x Optical Zoom, 720p HD video recording and 16 effective megapixels.

### 8.3. Evaluating the Level of Implementation of the LPS on the Study Case Project 

A total of 12 surveys were performed on two project managers, two field engineers, two technical office managers, two administrators, two safety supervisors, and two quality assurance engineers. The level of LPS implementation is shown in Table 4.

It was observed that the company’s initial implementation of the LPS was incomplete. It should be mentioned that collaborative safety planning sessions, five why analysis, and corrective measures were not entirely performed. In other words, the field engineers were not working with their support areas, especially, safety and health supervisors. This generated substandard acts and conditions that may be avoided if every worker in the project was aligned with safe and collaborative work. Thus, the missing LPS elements must be implemented. This project had an accumulated PPC of 81%, and even though this is a relatively high percentage, it may be affected by the incidents or accidents waiting to happen.

### 8.4. Work Sampling and Simultaneous Evaluation of Work Type and Safety Inspections

A work sampling was designed to achieve a minimum level of confidence of 95%, and a margin of error of 5%. The minimum number of samples needed for this purpose was 384 [22].

Four independent measurements were performed on 101 workers, obtaining 404 valid samples. The work type assessment and the safety inspections were executed simultaneously. The evaluations were video recorded. Table 5 shows the work sampling integrated with the safety classification.

Table 6 shows the summary of this evaluation and four video snapshots and their work classifications.

Corrective measures were taken, and a second assessment was performed to measure the improvement onsite. The obtained results were analyzed and shown in Table 7. 

### 8.5. Implementation of Safety and Production Corrective Measures

Corrective measures were given in the form of retraining for workers from point one forward. Therefore, the improvement of safety indicators was accomplished since there is a synergy in the simultaneous measurement of both. Table 8 shows the summary of this evaluation and the improvement in the acts and conditions.

It is important to mention that the company has now implemented all the elements of the LPS, meaning there are pull planning sessions, collaborative planning sessions, five why analyses, and corrective measures adoption. Moreover, the field engineers are working together with the support areas, including the safety supervisors, as a team. There were no major setbacks and everything went according to plan. What improved, ostensibly, were the health and safety indicators. 

According to the company data, the percentages of PW, CW, and NCW were normal. On the other hand, it can be observed in Table 6 and Table 8 that work with substandard acts decreased from 155 (38.4%) to 66 (16.3%). Further, it shows that work with substandard conditions decreased from 24 (5.94%) to 0%, among others. It is important to mention that training based on the staff’s behavior was reinforced. With this, the following measurements stayed within the standard conditions and the substandard acts were even further reduced. On the other hand, this project improved its weekly PPC to 86% and its accumulated PPC to 82%, which are similar values to the initial ones. However, the likelihood to have an incident or accident was reduced considerably. Thus, this will contribute to the safety costs in the mid and long term, and, most importantly, workers and third parties will be protected. Finally, and given the lack of explicit regulation, with a lean system, the civil and criminal responsibility of the involved agents would be covered in a better manner [59].

### 8.6. Statistical Correlation of Fatal, Serious, and Minor Accidents and Types of Work

For educational purposes, this study performed a simulated application of the methodology, considering that the percentages shown in Table 9 depict the representative measurements in a year.

For confidentiality reasons, the company did not provide its accident statistics. On the other hand, Peru does not count with official statistics for accident rate indicators that could be used to simulate a correlation with Peruvian average values [11,12]. Due to this, and solely for educational purposes, the 2017 official statistics of an important Peruvian company [60] will be used instead, in which a summary by accident is shown in Table 10. It is important to state that this company implements LPS in its building projects, so it is an excellent reference for our research. 

Then, Table 9 and Table 10 are statistically linked, and it is determined that for every 17 restricted work cases, there are 30 minor accidents, and the man hours are shown in Table 11.

**Figure 3:**
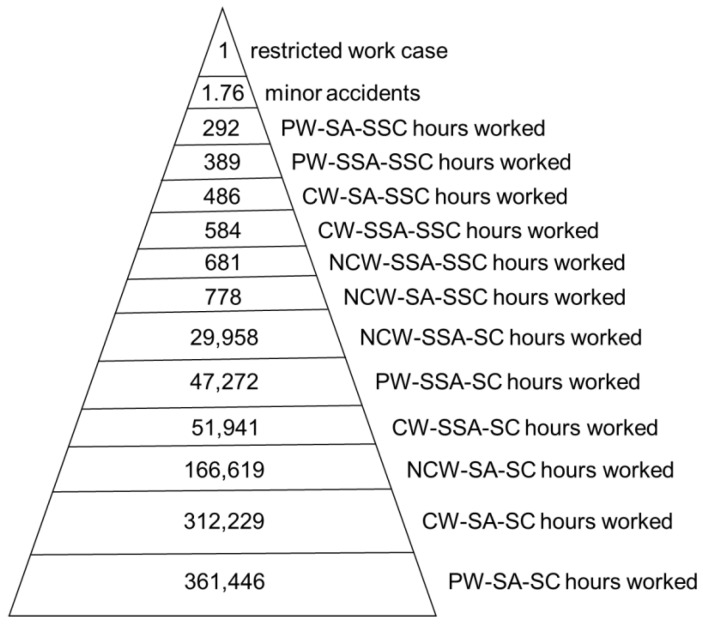
Correlation Models similar to the Heinrich model.

Based on this information it is possible to construct correlation models similar to the Heinrich model, selecting or grouping the variables as deemed pertinent. For instance, Figure 3 shows a model with the data from Table 10 and Table 11 divided by 17.

In addition, it is determined that the 3828 workers were exposed to a total of 54,567 h of substandard conditions (SSC), thus an average of 14.25 h of exposure per worker. It is also concluded that each worker conducted an average of 1605.20 h of the PW–SA–SC type of work in the year. In the same way, all the relations or indicators required are calculated, in addition to the conventional accident rates, making the correlation proposed in our research original, valuable, and easy to apply.

The proposed framework has the advantage that fewer resources will be used when making simultaneous measurements which are traditionally made separately. When analyzing these indicators in a collaborative environment, work satisfaction increases, which is very common with lean-approach projects. As demonstrated in the study, indicators were improved. However, work classifications and study results could vary according to the cultural level of the workers and professionals, their work habits, engineering and construction processes, industrialization level, and types of contracts, among other factors.

## 9. Conclusions

This paper presented an application that allowed the measurement of productive, contributory, and noncontributory work with substandard acts and conditions simultaneously in a construction site. In this manner, benchmarking was possible. 

The framework proposes a classification of work, measuring these indicators of production and safety simultaneously. Standard and substandard acts; standard and substandard conditions; and productive, contributory, and noncontributory work are statistically connected. To implement the proposed framework, the procedures of the production and the health and safety support areas must be updated, integrating the new approach.

As the case study showed, implementing the last planner system accordingly has an impact, not only on the productive but also on the health and safety indicators. This is accomplished since there is a synergy between the lean construction philosophy and the health and safety management systems. It presents evidence that respect for workers is fundamental to improving the health and safety indicators in construction projects. The behavior of workers, contractors, staff, and investors changed. 

Statistical correlations between occupational accidents and productive, contributory, and noncontributory work were obtained simultaneously by investing the least number of resources and, therefore, using a more economical method. The quality of the information was improved by obtaining integrated indicators, which reduced the uncertainty of making measurements separately, without a standard method, and with higher costs. The accidents by category and the classification of work are statistically connected in a simple way thanks to the framework proposed in this research.

This measurement system will allow the benchmarking with projects within the same company, and with other companies applying the same methodology. It is important to compare measurements in the same project phases.

In this research, we requested the express approval of the workers to be photographed and filmed according to Peruvian Law. However, when using other technologies, the legal analysis corresponding to every technology must be conducted.

A proposed future line of research is to automate the classification of the types of work, based on this classification, after gathering the visual information. The combination of several technologies such as sensors, radio-based or vision-based technologies, drones, etc., will present a real challenge.

## Figures and Tables

**Table 1:** Classification of production and safety work [11].

N°	Code	Production Work Classes	Act Classes	Condition Classes
1	PW-SA-SC	Productive Work	Standard Act	Standard Conditions
2	PW-SA-SSC	Productive Work	Standard Act	Substandard Conditions
3	PW-SSA-SC	Productive Work	Substandard Act	Standard Conditions
4	PW-SSA-SSC	Productive Work	Substandard Act	Substandard Conditions
5	CW-SA-SC	Contributory Work	Standard Act	Standard Conditions
6	CW-SA-SSC	Contributory Work	Standard Act	Substandard Conditions
7	CW-SSA-SC	Contributory Work	Substandard Act	Standard Conditions
8	CW-SSA-SSC	Contributory Work	Substandard Act	Substandard Conditions
9	NCW-SA-SC	Noncontributory Work	Standard Act	Standard Conditions
10	NCW-SA-SSC	Noncontributory Work	Standard Act	Substandard Conditions
11	NCW-SSA-SC	Noncontributory Work	Substandard Act	Standard Conditions
12	NCW-SSA-SSC	Noncontributory Work	Substandard Act	Substandard Conditions

**Table 2:** Results of the survey taken to classify productive, contributory, and noncontributory work.

Item	Activity	PW	CW	NCW	Total
1	Placement of horizontal reinforcement	325	9	0	334
2	Placement of vertical reinforcement	324	10	0	334
3	Placement of wire	246	84	4	334
4	Placement of slab form panel	273	54	7	334
5	Placement of formwork accessories in slab	225	103	6	334
6	Placement of wall form panel	267	64	3	334
7	Placement of formwork accessories in wall	205	127	2	334
8	Placing of formwork stiffeners	212	120	2	334
9	Placing formwork struts	223	107	4	334
10	Pouring concrete	316	16	2	334
11	Leveling concrete	212	122	0	334
12	Vibrating concrete	221	112	1	334
13	Installing electric pipes	307	24	3	334
14	Installing rectangular box	309	25	0	334
15	Wall grinding	260	70	4	334
16	Leveling mortar	211	120	3	334
17	Plastering walls	226	106	2	334
18	Installing gas pipes	307	25	2	334
19	Fusing water pipes	210	118	6	334
20	Installation of sewage pipeline	285	48	1	334
21	Brick placement	288	44	2	334
22	Filling	274	57	3	334
23	Placing wire in masonry walls	179	151	4	334
24	Laying out	167	159	8	334
25	Sanding door frame	194	118	22	334
26	Installing window frame	260	69	5	334
27	Installing drywall profiles	268	64	2	334
28	Installing ceramic tiles	293	40	1	334
29	Placing telecommunications cable	256	76	2	334
30	Installing hinges on door	260	73	1	334
31	Plastering drywall	271	61	2	334
32	Filling door frame	232	95	7	334
33	Installing wallpaper	260	63	11	334
34	Placing props in lightened slab formwork	216	112	6	334
35	Placing beams in lightened slab formwork	240	92	2	334
36	Placing plywood in lightened slab formwork	235	96	3	334
37	Nailing components in lightened slab formwork	166	162	6	334
38	Adjusting beams in lightened slab formwork	180	149	5	334
39	Placing precast joist	296	38	0	334
40	Adjusting precast joist	247	86	1	334
41	Placing bricks for lightened slab	290	42	2	334
42	Installing Styrofoam to seal lightened slab bricks	212	117	5	334
43	Join water pipes	213	119	2	334
44	Installing water pipes	312	22	0	334
45	Installing pipe fittings and connectors	289	45	0	334
46	Finding materials	42	204	88	334
47	Placing separators for concrete	101	196	37	334
48	Moving to another point to install reinforcement	16	209	109	334
49	Measuring	41	271	22	334
50	Opening reinforcement packages with a shear	8	262	64	334
51	Transporting material	24	279	31	334
52	Receiving/Giving instructions	20	261	53	334
53	Removing wall formwork accessories	129	193	12	334
54	Removing formwork aligner	110	213	11	334
55	Carrying tools	11	272	51	334
56	Removing plywood in wall formwork	121	204	9	334
57	Finding accessories of slab formwork	6	212	116	334
58	Removing accessories of slab formwork	109	213	12	334
59	Removing plywood in slab formwork	130	193	11	334
60	Applying mold release agent	146	174	14	334
61	Cleaning formwork	37	257	40	334
62	Level out concrete with a shovel or foot	119	189	26	334
63	Carrying material	40	270	24	334
64	Placing baseboard on one side	165	167	2	334
65	Giving/Receiving instructions	37	262	35	334
66	Gathering concrete from the slab to use it in the parapet	79	226	29	334
67	Removing concrete from formwork using a hammer	34	259	41	334
68	Placing/removing separators for concrete	38	278	18	334
69	Cleaning tools	3	242	89	334
70	Cleaning the work area, a day before	15	250	69	334
71	Removing equipment from the work area	6	235	93	334
72	Maneuvering mixer or pump to pour concrete	112	211	11	334
73	Maneuvering pipes, hoses, and accessories	112	210	12	334
74	Pouring leftover concrete from a slab in a wall	95	156	83	334
75	Cutting electrical pipes	118	207	9	334
76	Cutting gas pipes	111	213	10	334
77	Repairing walls to install pipes	127	123	84	334
78	Introducing cable guides in electrical pipes	168	163	3	334
79	Cutting sewage pipes	95	229	10	334
80	Preparing materials	199	125	10	334
81	Wet wall for masonry work	76	247	11	334
82	Placing accessories in wall	123	200	11	334
83	Assembly of scaffold	59	264	11	334
84	Water leak test	95	224	15	334
85	Measuring	54	256	24	334
86	Using lifeline	38	275	21	334
87	Preparing mortar	195	129	10	334
88	Measuring	42	272	20	334
89	Transporting cleaning materials	15	254	65	334
90	Performing a hydrostatic test	102	214	18	334
91	Installing safety rails	64	245	25	334
92	Drilling wall for plastering	124	186	24	334
93	Cutting profiles for drywall	157	171	6	334
94	Cutting tiles	86	240	8	334
95	Cutting wallpaper	67	245	22	334
96	Preparing workspace	67	239	28	334
97	Drilling on demolition debris to make a trench	148	170	16	334
98	To shovel	126	167	41	334
99	Transporting objects	29	274	31	334
100	Transporting materials with a crane	26	282	26	334
101	Verifying the alignment of the ceiling	59	255	20	334
102	Set up cutting machine	32	271	31	334
103	Drilling on melamine cabinets	101	210	23	334
104	Drilling a slab	119	185	30	334
105	Repairing a slab	75	144	115	334
106	Training the crew on safety during construction	44	251	39	334
107	Receiving safety training	35	261	38	334
108	Receiving a safety induction because of a substandard act	28	200	106	334
109	Installing collective protection equipment	59	243	32	334
110	Safety signs	57	245	32	334
111	Safety drill	32	257	45	334
112	Level out	158	174	2	334
113	Cutting water pipes	110	217	7	334
114	Measuring onsite	89	232	13	334
115	Reading blueprints	57	253	24	334
116	Running QA tests (pressure or water tightness)	113	200	21	334
117	Preventive maintenance for equipment	35	264	35	334
118	Corrective maintenance for equipment	42	208	84	334
119	Going to/coming back from lunch out of schedule	13	72	249	334
120	Waiting for the concrete bucket to pour concrete	14	90	230	334
121	Transporting an empty bucket	7	112	215	334
122	Waiting	1	76	257	334
123	Redoing work (Straightening steel reinforcement)	4	90	240	334
124	Idle time	2	75	257	334
125	Going to the toilette	1	129	204	334
126	Walking empty handed	4	71	259	334
127	Having breakfast	13	119	202	334
128	Redoing work	17	83	234	334

**Table 3:** Groups of activities result from the survey.

Item	Activity	Work Type	Code
1	Alignment	PW	PW01
2	Application of materials	PW	PW02
3	Filling	PW	PW03
4	Fusion of water pipes	PW	PW04
5	Installation	PW	PW05
6	Leveling	PW	PW06
7	Placement of materials	PW	PW07
8	Placement of formwork	PW	PW08
9	Plastering	PW	PW09
10	Preparation of material	PW	PW10
11	Sand door frame	PW	PW11
12	Vibrating concrete	PW	PW12
13	Wiring	PW	PW13
14	Applying mold release agent	CW	CW01
15	Assembly of scaffold	CW	CW02
16	Carrying material or tools	CW	CW03
17	Cleaning	CW	CW04
18	Construction site inspection	CW	CW05
19	Cutting	CW	CW06
20	Demarcation of work area	CW	CW07
21	Drilling	CW	CW08
22	Enable cutting machine	CW	CW09
23	Giving instructions	CW	CW10
24	Installation of prevention measures	CW	CW11
25	Lifting equipment with a crane	CW	CW12
26	Maneuvering pipes or accessories to productive work	CW	CW13
27	Moving materials	CW	CW14
28	Patching wall to install tiles or pipes	CW	CW15
29	Placement of equipment	CW	CW16
30	Preparing workspace	CW	CW17
31	Receiving instructions	CW	CW18
32	Removing	CW	CW19
33	Safety supervision	CW	CW20
34	Shoveling	CW	CW21
35	Taking measurements	CW	CW22
36	Test	CW	CW23
37	Verifying	CW	CW24
38	Wet wall for masonry works	CW	CW25
39	Having breakfast	NCW	NCW01
40	Idle time	NCW	NCW02
41	Redoing work	NCW	NCW03
42	Standing worker	NCW	NCW04
43	Transporting an empty bucket	NCW	NCW05
44	Unproductive trip	NCW	NCW06
45	Using the restroom	NCW	NCW07
46	Waiting	NCW	NCW08

**Table 4:** Initial Level of implementation of the LPS of the Project.

Last Planner System Element	Implementation Rate
Master plan	100%
Health and safety plan	100%
Pull Planning Session	100%
Lookahead	100%
Percent Plan Completion (PPC)	100%
Collaborative Safety Planning	50%
5 Why Analysis and Corrective Measures	25%

**Table 5:** Work Sampling based on the new classification.

Activity	Number	Type of Work	Type of Act	Type of Condition	Classification
Brick placement	3	PW	SA	SC	PW-SA-SC
Brick placement	2	PW	SSA	SC	PW-SSA-SC
Filling door frame	4	PW	SSA	SC	PW-SSA-SC
Fusing water pipes	2	PW	SSA	SC	PW-SSA-SC
Installation of elevator	3	PW	SA	SC	PW-SA-SC
Installation of elevator	2	PW	SSA	SC	PW-SSA-SC
Installing ceramic tiles	2	PW	SSA	SC	PW-SSA-SC
Installing electric pipes	9	PW	SA	SC	PW-SA-SC
Installing electric pipes	2	PW	SSA	SC	PW-SSA-SC
Installing gas pipes	3	PW	SA	SC	PW-SA-SC
Installing hinges on door	2	PW	SSA	SC	PW-SSA-SC
Installing horizontal reinforcement	3	PW	SA	SC	PW-SA-SC
Installing profiles for drywall	2	PW	SSA	SC	PW-SSA-SC
Installing rectangular boxes	3	PW	SA	SC	PW-SA-SC
Installing sewage pipes	2	PW	SSA	SC	PW-SSA-SC
Installing wallpaper	2	PW	SSA	SC	PW-SSA-SC
Installing window frame	3	PW	SA	SC	PW-SA-SC
Introducing cable guides in electrical pipes	3	PW	SA	SC	PW-SA-SC
Introducing cable guides in electrical pipes	6	PW	SSA	SC	PW-SSA-SC
Level out concrete	6	PW	SA	SC	PW-SA-SC
Level out concrete	3	PW	SSA	SSC	PW-SSA-SSC
Level out concrete with a shovel or foot	2	PW	SSA	SC	PW-SSA-SC
Placement of wire	18	PW	SA	SC	PW-SA-SC
Placement of wire	4	PW	SSA	SC	PW-SSA-SC
Placing accessories in wall formwork	6	PW	SA	SC	PW-SA-SC
Placing plywood in wall formwork	3	PW	SA	SC	PW-SA-SC
Placing telecommunications cable	2	PW	SSA	SC	PW-SSA-SC
Plastering drywall	2	PW	SSA	SC	PW-SSA-SC
Plastering wall	3	PW	SA	SC	PW-SA-SC
Plastering wall	2	PW	SSA	SC	PW-SSA-SC
Preparing electric material	2	PW	SSA	SC	PW-SSA-SC
Preparing material to install pipes	3	PW	SA	SC	PW-SA-SC
Preparing melamine cabinets	2	PW	SSA	SC	PW-SSA-SC
Preparing mortar	2	PW	SSA	SC	PW-SSA-SC
Sanding door frame	3	PW	SA	SC	PW-SA-SC
Sanding door frame	2	PW	SSA	SC	PW-SSA-SC
Vibrating concrete	2	PW	SSA	SC	PW-SSA-SC
Wall grinding	6	PW	SA	SC	PW-SA-SC
Wall grinding	8	PW	SSA	SC	PW-SSA-SC
Applying mold release agent	2	CW	SA	SC	CW-SA-SC
Carrying tools	2	CW	SA	SC	CW-SA-SC
Cleaning	14	CW	SA	SC	CW-SA-SC
Drilling on demolition debris	2	CW	SA	SC	CW-SA-SC
Drilling on melamine cabinets	2	CW	SSA	SC	CW-SSA-SC
Erect scaffolding	2	CW	SA	SC	CW-SA-SC
Giving instructions	2	CW	SA	SC	CW-SA-SC
Giving instructions	2	CW	SSA	SC	CW-SSA-SC
Going to the toilette	2	CW	SA	SC	CW-SA-SC
Installing safety rails	2	CW	SA	SSC	CW-SA-SSC
Using scaffolding	1	CW	SA	SSC	CW-SA-SSC
Laying out	2	CW	SA	SC	CW-SA-SC
Measuring	2	CW	SA	SC	CW-SA-SC
Measuring	10	CW	SSA	SC	CW-SSA-SC
Preparing workspace	2	CW	SA	SC	CW-SA-SC
Receiving instructions	4	CW	SA	SC	CW-SA-SC
Receiving instructions	2	CW	SSA	SC	CW-SSA-SC
Removing accessories in wall formwork	2	CW	SA	SC	CW-SA-SC
Removing accessory	2	CW	SA	SC	CW-SA-SC
Removing formwork struts	2	CW	SA	SC	CW-SA-SC
Removing plywood in wall formwork	3	CW	SA	SSC	CW-SA-SSC
Removing plywood in wall formwork	4	CW	SA	SC	CW-SA-SC
Removing plywood in wall formwork	6	CW	SSA	SC	CW-SSA-SC
Risk Prevention	2	CW	SA	SC	CW-SA-SC
Risk prevention—Securing lifeline	2	CW	SSA	SC	CW-SSA-SC
Running hydrostatic test	2	CW	SSA	SC	CW-SSA-SC
Set up cutting machine	2	CW	SSA	SC	CW-SSA-SC
Shoveling	4	CW	SA	SC	CW-SA-SC
Transporting material	36	CW	SA	SC	CW-SA-SC
Transporting material	8	CW	SSA	SC	CW-SSA-SC
Transporting materials with a crane	2	CW	SSA	SC	CW-SSA-SC
Transporting objects	8	CW	SA	SC	CW-SA-SC
Transporting objects	4	CW	SSA	SC	CW-SSA-SC
Verifying before wall grinding	2	CW	SA	SC	CW-SA-SC
Verifying the alignment of the ceiling	2	CW	SSA	SC	CW-SSA-SC
Water leak test	2	CW	SA	SC	CW-SA-SC
Wet wall for masonry works	2	CW	SA	SC	CW-SA-SC
Idle time	15	NCW	SA	SC	NCW-SA-SC
Idle time	34	NCW	SSA	SC	NCW-SSA-SC
Idle time	9	NCW	SA	SSC	NCW-SA-SSC
Redone work—Drilling on slab	3	NCW	SA	SSC	NCW-SA-SSC
Redone work—Drilling on slab	3	NCW	SA	SC	NCW-SA-SC
Redone work—Drilling wall	3	NCW	SA	SC	NCW-SA-SC
Repairing slab	6	NCW	SA	SC	NCW-SA-SC
Standing worker	12	NCW	SA	SC	NCW-SA-SC
Standing worker	4	NCW	SSA	SC	NCW-SSA-SC
Standing worker	3	NCW	SA	SSC	NCW-SA-SSC
Transporting an empty bucket	2	NCW	SSA	SC	NCW-SSA-SC
Unproductive trip	9	NCW	SA	SC	NCW-SA-SC
Unproductive trip	10	NCW	SSA	SC	NCW-SSA-SC

**Table 6:** Application of new classification and video snapshots.

Code	Number	Percentage
PW-SA-SC	78	19.3%
PW-SA-SSC	0	0%
PW-SSA-SC	58	14.4%
PW-SSA-SSC	3	0.7%
CW-SA-SC	102	25.3%
CW-SA-SSC	6	1.5%
CW-SSA-SC	44	10.9%
CW-SSA-SSC	0	0%
NCW-SA-SC	48	11.9%
NCW-SA-SSC	15	3.7%
NCW-SSA-SC	50	12.4%
NCW-SSA-SSC	0	0%

**Table 7:** Work sampling based on the new classification after corrective measures.

Activity	Number	Type of Work	Type of Act	Type of Condition	Classification
Installing electric pipes	17	PW	SA	SC	PW-SA-SC
Installing electric pipes	1	PW	SSA	SC	PW-SSA-SC
Installing gas pipes	5	PW	SA	SC	PW-SA-SC
Installing gas pipes	6	PW	SSA	SC	PW-SSA-SC
Installing reinforcement	15	PW	SA	SC	PW-SA-SC
Installing reinforcement	1	PW	SSA	SC	PW-SSA-SC
Installing sewage pipeline	2	PW	SA	SC	PW-SA-SC
Installing sewage pipeline	3	PW	SSA	SC	PW-SSA-SC
Installing water pipes	4	PW	SA	SC	PW-SA-SC
Installing water pipes	8	PW	SSA	SC	PW-SSA-SC
Level out concrete	1	PW	SA	SC	PW-SA-SC
Placement of formwork	34	PW	SA	SC	PW-SA-SC
Placement of formwork	1	PW	SSA	SC	PW-SSA-SC
Placing separators for concrete	5	PW	SA	SC	PW-SA-SC
Placing struts in formwork	9	PW	SA	SC	PW-SA-SC
Placing wire	15	PW	SA	SC	PW-SA-SC
Placing wire	3	PW	SSA	SC	PW-SSA-SC
Pouring concrete	4	PW	SA	SC	PW-SA-SC
Preparing material	13	PW	SA	SC	PW-SA-SC
Preparing material	2	PW	SSA	SC	PW-SSA-SC
Preparing reinforcement	3	PW	SA	SC	PW-SA-SC
Vibrating concrete	3	PW	SA	SC	PW-SA-SC
Applying mold release agent	1	CW	SA	SC	CW-SA-SC
Assemble scaffolding	5	CW	SA	SC	CW-SA-SC
Cleaning	15	CW	SA	SC	CW-SA-SC
Cleaning	4	CW	SSA	SC	CW-SSA-SC
Cleaning formwork	1	CW	SA	SC	CW-SA-SC
Cleaning reinforcement	2	CW	SA	SC	CW-SA-SC
Giving instructions	8	CW	SA	SC	CW-SA-SC
Giving instructions	5	CW	SSA	SC	CW-SSA-SC
Laying out	3	CW	SSA	SC	CW-SSA-SC
Measuring	17	CW	SA	SC	CW-SA-SC
Measuring	11	CW	SSA	SC	CW-SSA-SC
Posting signs	1	CW	SSA	SC	CW-SSA-SC
Transporting formwork	2	CW	SA	SC	CW-SA-SC
Transporting formwork	2	CW	SSA	SC	CW-SSA-SC
Transporting objects	1	CW	SA	SC	CW-SA-SC
Preparing work area to pour concrete	2	CW	SA	SC	CW-SA-SC
Receiving instructions	12	CW	SA	SC	CW-SA-SC
Receiving instructions	5	CW	SSA	SC	CW-SSA-SC
Removing struts	2	CW	SA	SC	CW-SA-SC
Transporting material	48	CW	SA	SC	CW-SA-SC
Transporting material	4	CW	SSA	SC	CW-SSA-SC
Transporting materials with a crane	9	CW	SA	SC	CW-SA-SC
Transporting objects	30	CW	SA	SC	CW-SA-SC
Transporting scaffolding	2	CW	SA	SC	CW-SA-SC
Unloading joists	3	CW	SA	SC	CW-SA-SC
Idle time	9	NCW	SA	SC	NCW-SA-SC
Idle time	1	NCW	SSA	SC	NCW-SSA-SC
Redone work—Drilling	1	NCW	SA	SC	NCW-SA-SC
Unproductive trip	4	NCW	SA	SC	NCW-SA-SC
Unproductive trip	3	NCW	SSA	SC	NCW-SSA-SC
Waiting	26	NCW	SA	SC	NCW-SA-SC
Waiting	2	NCW	SSA	SC	NCW-SSA-SC
Wall grinding	8	NCW	SA	SC	NCW-SA-SC

**Table 8:** Improvement in production and safety.

Code	Number	Percentage
PW-SA-SC	130	32.2%
PW-SA-SSC	0	0%
PW-SSA-SC	25	6.2%
PW-SSA-SSC	0	0%
CW-SA-SC	160	39.6%
CW-SA-SSC	0	0%
CW-SSA-SC	35	8.7%
CW-SSA-SSC	0	0%
NCW-SA-SC	48	11.9%
NCW-SA-SSC	0	0%
NCW-SSA-SC	6	1.5%
NCW-SSA-SSC	0	0%

**Table 9:** Percentages of measurements in a year.

Code	Percentage
PW-SA-SC	37.16%
PW-SA-SSC	0.03%
PW-SSA-SC	4.86%
PW-SSA-SSC	0.04%
CW-SA-SC	32.10%
CW-SA-SSC	0.05%
CW-SSA-SC	5.34%
CW-SSA-SSC	0.06%
NCW-SA-SC	17.13%
NCW-SA-SSC	0.08%
NCW-SSA-SC	3.08%
NCW-SSA-SSC	0.07%

**Table 10:** Accident rate of a Peruvian construction company [60].

Indicator	2017
Number of man hours	16,535,491
Number of workers	3828
Number of restricted work case	17
Number of minor accidents	30
Number of fatal accidents	0
Working days lost due to accidents	450

**Table 11:** Hours worked in a year.

Code	Percentage	Hours Worked
PW-SA-SC	37.16%	6,144,588
PW-SA-SSC	0.03%	4961
PW-SSA-SC	4.86%	803,625
PW-SSA-SSC	0.04%	6614
CW-SA-SC	32.10%	5,307,893
CW-SA-SSC	0.05%	8268
CW-SSA-SC	5.34%	882,995
CW-SSA-SSC	0.06%	9921
NCW-SA-SC	17.13%	2,832,530
NCW-SA-SSC	0.08%	13,228
NCW-SSA-SC	3.08%	509,293
NCW-SSA-SSC	0.07%	11,575
	100%	16,535,491

## Data Availability

The data used in this study are available on Table 1, Table 2, Table 3, Table 4, Table 5, Table 6, Table 7, Table 8, Table 9, Table 10 and Table 11.
